# Energy-Efficient Collaborative Outdoor Localization for Participatory Sensing

**DOI:** 10.3390/s16060762

**Published:** 2016-05-25

**Authors:** Wendong Wang, Teng Xi, Edith C.-H. Ngai, Zheng Song

**Affiliations:** 1 State Key Laboratory of Networking and Switching Technology, Beijing University of Posts and Telecommunications, XiTuCheng Road 10#, Haidian District, Beijing 100876, China; wdwang@bupt.edu.cn; 2 Department of Information Technology, Uppsala University, Uppsala 751 05, Sweden; edith.ngai@it.uu.se; 3 Department of Computer Science, Virginia Tech, Blacksburg, VA 24060, USA; sonyyt@gmail.com

**Keywords:** participatory sensing, collaborative localization, device to device localization, energy efficiency

## Abstract

Location information is a key element of participatory sensing. Many mobile and sensing applications require location information to provide better recommendations, object search and trip planning. However, continuous GPS positioning consumes much energy, which may drain the battery of mobile devices quickly. Although WiFi and cell tower positioning are alternatives, they provide lower accuracy compared to GPS. This paper solves the above problem by proposing a novel localization scheme through the collaboration of multiple mobile devices to reduce energy consumption and provide accurate positioning. Under our scheme, the mobile devices are divided into three groups, namely the broadcaster group, the location information receiver group and the normal participant group. Only the broadcaster group and the normal participant group use their GPS. The location information receiver group, on the other hand, makes use of the locations broadcast by the broadcaster group to estimate their locations. We formulate the broadcaster set selection problem and propose two novel algorithms to minimize the energy consumption in collaborative localization. Simulations with real traces show that our proposed solution can save up to 68% of the energy of all of the participants and provide more accurate locations than WiFi and cellular network positioning.

## 1. Introduction

A participatory sensing system [1] supports a large number of mobile users to collect sensor data and share the information of their environment. Many sensor data and mobile services are location oriented. For example, a driver may want to know the gas price of nearby gas stations or the traffic conditions around a specific area. Potential applications of location-based services in participatory sensing range from personal healthcare, object search to entertainment. However, obtaining location information often requires large energy consumption, which may discourage many mobile participants. Given the limited battery life of a mobile device, it is crucial to minimize the energy consumption in localization.

GPS localization is known as a major source of energy consumption in mobile devices. Continuous GPS sensing can easily drain the battery of a mobile device in five to six hours [2]. However, many participatory sensing applications require the mobile devices to turn on their GPS to collect location information. Even though alternative localization methods are available, such as cell-tower-based localization and WiFi-based localization, they fail to provide the same level of accuracy as the GPS.

The traditional approach consumes much energy, as each mobile device has to turn on its GPS continuously to collect and report sensor and location data to the server. To address this problem, we propose a novel approach of collaborative localization, which suggests that mobile devices share location information with other devices in the same vicinity. Apart from sharing GPS readings, we leverage lightweight sensors, such as the accelerometer and gyroscope, to approximate the location of individual devices in collaborative localization. These lightweight sensors can run for more than 20 h continuously on a smartphone [3], which can assist localization and reduce energy consumption.

Different from the traditional approach, our proposed approach divides the mobile devices into three categories, the broadcasters, the location information receivers (LIRs) and the normal participants. All members in the three categories collect sensor data periodically, but the ways that they obtain location data and label sensor data are different. The broadcasters and the normal participants turn on their GPS to obtain their location data, which are then used to label their sensor data. To save energy, the LIRs do not turn on their GPS. Instead, they estimate their locations according to the location information broadcast by the surrounding broadcasters.

Each mobile device will be assigned to a category by the server, and its category will be updated from time to time. We formulate the broadcaster set selection problem (BSSP) to select the set of broadcasters and to determine how long the selected mobile devices will be broadcasters. The LIRs will use the location data from the broadcasters nearby to estimate their own locations. The remaining stand-alone mobile participants are the normal participants, who use GPS to obtain location data as in the traditional approach. The objective of BSSP is to minimize the overall energy consumption of all of the participants.

The contributions of this paper are summarized as follows:

- A collaborative localization architecture is proposed, which enables flexible adjustment on location accuracy based on the application requirement. A mathematical model is set up to measure the total energy consumption of mobile participants.
- The operations in collaborative localization are designed and presented, which are feasible to be implemented for participatory sensing systems.
- The BSSP is formulated, which aims to minimize the energy consumption of all of the participants.
- Two novel heuristic algorithms are proposed to solve the BSSP.
- The performance of the proposed solution has been evaluated thoroughly by simulations using real mobility traces. The results show that our proposed algorithms can save up to 68% of the total energy in localization.

A preliminary conference paper based on this work can be found in [4]. The rest of the paper is organized as follows. Section 2 presents the related work. Section 3 shows the system architecture and information flows. Section 4 shows the energy model and formulates the BSSP. Section 5 describes our proposed broadcaster set selection algorithms. Section 6 evaluates the performance of our proposed algorithms by simulations using real mobility traces. Finally, Section 7 concludes the paper.

## 2. Related Work

There are several types of mobile sensing, including participatory sensing, opportunistic sensing and crowdsensing [5]. The key idea of mobile sensing systems [5] is to have ordinary citizens collect and share sensor data from their surrounding environment by using their smartphones. For example, the Common Sense project [6] has developed a participatory sensing system that allows mobile participants to measure their personal exposure to air pollution. Similarly, Kanjo [7] has shown a system that allows participants to collect and share the noise pollution information. Zhang [8] *et al.* have proposed a new cosine theorem-based method for identifying and expressing conflicting data, which can be used for fusing conflicting data collected by participants.

In addition, research work has been done to address the participant selection problem in mobile sensing. Tuncay *et al.* [9] have exploited the user behaviors and selected the participants based on their mobility history. However, this approach relies heavily on the knowledge of participant trajectories, which increases the risk of leaking user privacy. Reddy *et al.* [10] have developed a selection framework that enables organizers to choose well-suited participants to collect data. Similarly, Song *et al.* [11] have developed a participant selection framework to satisfy the quality-of-information (QoI) requirements of the sensing tasks. Other than that, Lu *et al.* [12] have studied initiating sampling around a specific location. However, all of the above schemes could be further improved by taking energy saving into consideration.

Energy saving is a very important issue in mobile sensing systems. One common approach is to reduce the sampling rates, though this may lead to lower accuracy of the sensing results [13,14]. Other approaches utilize additional sensors, such as accelerometers and orientation sensors, to assist localization [15]. However, none of the above works have explored the possibility of sharing location information with neighboring devices.

Different from the traditional approach using GPS, alternative localization methods have been exploited. For example, Johnson and Seeling [16] have proposed a scheme based on Bluetooth device names to enable power-optimized *ad hoc* localization of mobile devices. However, this work mainly focused on the naming scheme, while the optimization of energy consumption during localization remains to be further explored. Other than that, some authors have suggested to use location beacons as localization references [17], but this method requires either fixed or mobile beacons. Similarly, Zhang and Yu [18] have proposed a beacon selection method that selects equilateral triangle nodes to be beacons. However, the location beacons may limit the energy saving performance and increase the deployment and maintenance cost.

Different from the above work, we aim to minimize the energy consumption through collaborative localization without deploying any beacon nodes. Besides, we only require users’ current locations instead of any historical trajectories; thus, our approach can protect user privacy.

## 3. System Architecture and Information Flow

In this section, we first illustrate the system architecture for the participatory sensing system and then describe the information flows of collaborative localization.

### 3.1. Motivating Scenario for Collaborative Localization

**Figure 1:**
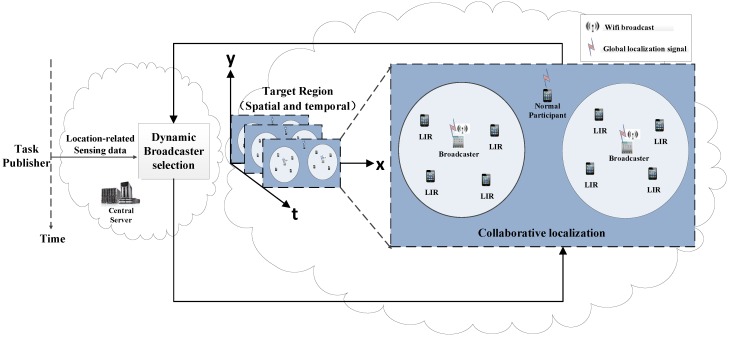
System architecture.

We consider a participatory sensing system in Figure 1. It consists of a central server, a task publisher and a set of *M* smartphone users, M≜{1,2,...,M}, in the region. The task publisher sends the sensing task to the server. The server then forwards the task to all of the participants.

We divide the length of a sensing task into a set of time slots, denoted by $T=\{t_{s},t_{s}+1,...,t_{e}\}$, where $t_{s}$ and $t_{e}$ are the start time and the end time of the sensing task. Based on the participants’ current locations, the server will assign different roles to the participants to perform the sensing and localization. The roles will last for a certain period of time, then the server will dynamically assign new roles to the participants based on their updated locations.

All of the participants in our system are divided into three different roles. The first role is the broadcaster, which broadcasts its location and movement information to the surrounding participants. The set of broadcasters during $\left[t_{1},t_{2}\right]$ is denoted by $B^{t_{1}t_{2}}$, $\forall t_{1},t_{2}$∈T. The second role is the location information receiver (LIR), which relies on the broadcasters’ locations to calculate its own location. The third role is the normal participant, which does not receive any location broadcast from the surrounding. Hence, it has to turn on its GPS to obtain the location. The set of normal participants during $\left[t_{1},t_{2}\right]$ is denoted by $N^{t_{1}t_{2}}$, $\forall t_{1},t_{2}$∈T.

Table 1 lists the frequently-used notations in the paper.

### 3.2. Information Flow

After the task publisher publishes the sensing task, the server will send the task to all participants. The participants will register their locations to the server. In the initialization phase, all participants turn on their GPS to obtain their initial locations. Based on the participants’ locations, the server will assign different roles to the participants and how long the roles will last, which is denoted by *l*.

During each collaborative localization time period, all participants collect sensor data, but they label the location information of the collected data differently. The broadcasters and the normal participants turn on their GPS to obtain their locations. Besides, the broadcasters also broadcast their locations and their movement information periodically to their surrounding LIRs. Based on the received broadcasts, the surrounding LIRs can calculate their locations using the device to device localization method proposed in our previous work [19]. At the end of the collaborative localization period, all participants upload the collected sensor data and their locations to the server through the cellular network. Based on the participants’ current locations, the server will choose and inform their new roles for the next collaborative localization period and how long this period will last.

**Figure 2:**
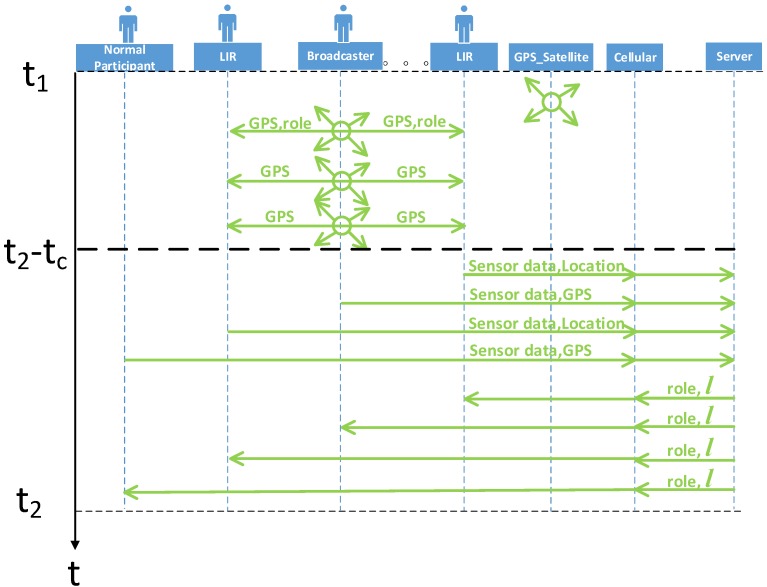
The collaborative localization during [$t_{1},t_{2}$].

Figure 2 shows the collaborative localization during [$t_{1},t_{2}$]. Let $t_{c}$ be the time that the participants need for sending data and receiving the roles. During [$t_{1},t_{2}-t_{c}$], the broadcasters and the normal participants obtain their locations from their GPS, so that they can label the locations of the collected sensor data directly. On the contrary, the LIRs rely on the broadcasters’ locations to calculate their locations and label the sensor data. During [$t_{2}-t_{c},t_{2}$], all participants upload the collected sensor data and their labeled locations to the server and wait for the server’s reply. Although all participants start sending at the same time, due to jitter or latency, the collected sensor data will arrive in random order on the server side. There is a time window for the server to receive the data. Similarly, there is a time window for the participants to receive the roles.

**Figure 3:**
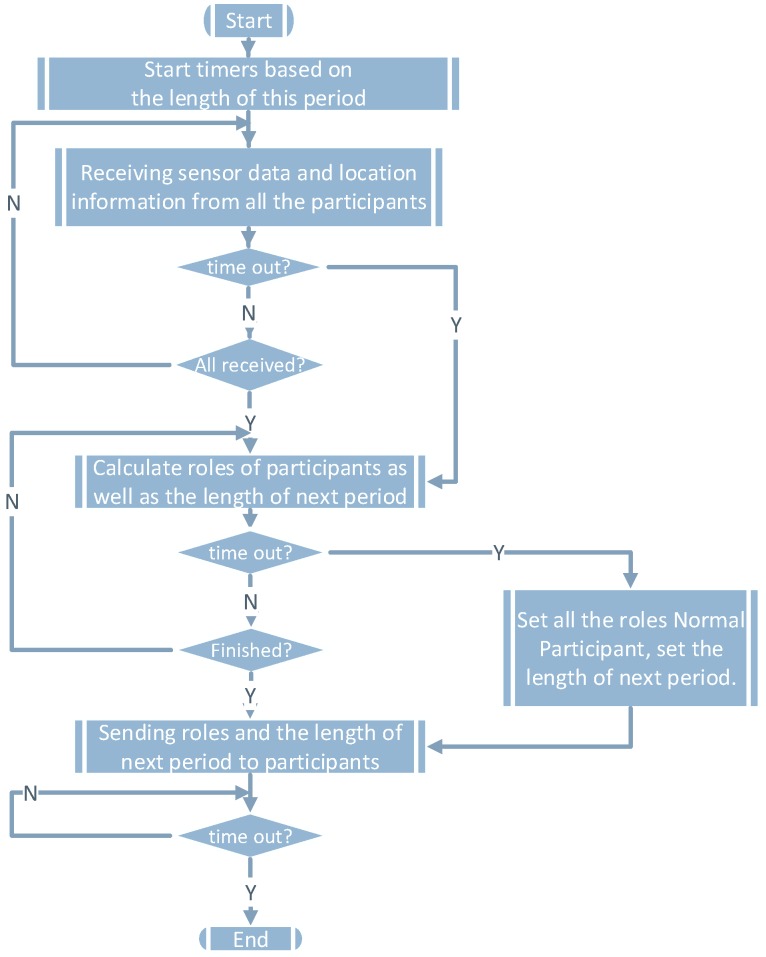
The server’s function flow.

Figure 3 shows the corresponding function flow of the server. During [$t_{2}-t_{c},t_{2}$], the server will decide on the roles of all participants in the next collaborative localization period and the length of the period. The goal is to minimize the power of all participants. Finally, the server will send the roles and the length of the next period to all participants. If the server does not receive sensor data from certain participants before timing out, the server will set the role of those participants as normal participants.

## 4. Modeling and Problem Formulation

In this section, we will present device to device localization. Then, we will model the energy consumption of different roles in our system. Finally, we will formulate the BSSP.

### 4.1. Device to Device Localization

**Figure 4:**
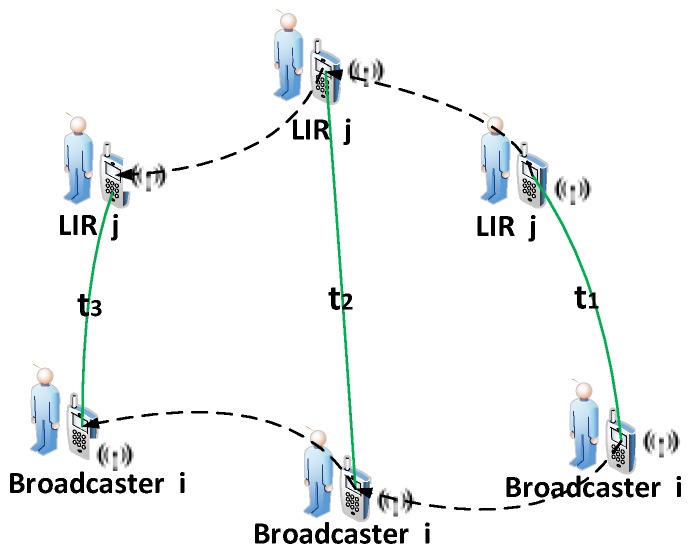
Device to device localization.

This subsection shows how an LIR *j* can calculate its location through the WiFi broadcast of a broadcaster *i*, where ∀i,j∈M. Locations in the real world are measured in three dimensions, namely latitude, altitude and longitude. Without loss of generality and for simplicity, a two-dimensional scenario without the altitude dimensional is considered in this paper. As shown in Figure 4, the scenario comprises two mobile devices held by the mobile users, *i* and *j*. The arrows indicate the trajectories of broadcaster *i* and LIR *j*.

The basic procedure of device to device localization can be divided into three phases, time stamped by $t_{1}$, $t_{2}$, $t_{3}$, where $\forall t_{1},t_{2},t_{3}$∈T and $t_{1}<t_{2}<t_{3}$.

As mentioned before, broadcaster *i* broadcasts its location periodically. $t_{1}$ is the time when LIR *j* first receives *i*’s broadcast. Then, LIR *j* receives *i*’s broadcast the second and third times at $t_{2}$ and $t_{3}$. The RSSI between *i* to *j* at time tis denoted by $r_{ij}^{t}$. The movement of participant *i* from $t_{1}$ to $t_{2}$, in terms of the walking distance and heading angle, is denoted by a vector ${\vec{m}}_{i}^{t_{1}t_{2}}$.

The relative movement between *i* and *j* from $t_{1}$ to $t_{2}$ is denoted by ${\vec{m}}_{ij}^{t_{1}t_{2}}$. The relative location of the LIR *j* towards the broadcaster *i* can be calculated as follows.

The movement measurement is based on the step detection method, which is commonly used in pedestrian localization [20]. The pedestrian’s walking steps are detected by the acceleration signal, and the step length is estimated based on the statistical model. In each step, the walking direction is measured by the digital compass on the mobile devices. The nonlinear model [21] is selected to estimate the step length. The step length $L^{t_{1}t_{2}}$ of pedestrians during [$t_{1}$,$t_{2}$] is determined by his accelerator readings:

(1) $$L^{t_{1}t_{2}}=K*\sqrt[{4}]{A_{\max}^{t_{1}t_{2}}-A_{\min}^{t_{2}t_{2}}}\;$$

where $A_{\max}^{t_{1}t_{2}}$ represents the maximum acceleration during [$t_{1}$,$t_{2}$], $A_{\min}^{t_{1}t_{2}}$ represents the minimum acceleration during [$t_{1}$,$t_{2}$] and *K* is the coefficient.

Based on the counted number of steps, the estimated step length, the direction of each step and the movement vector of LIR *j* can be calculated separately by:

(2) $$\begin{array}{c} {\vec{m}}_{j}^{t_{1}t_{2}}.x=\sum_{k=1}^{{\mathrm{StepNum}}^{t_{1}t_{2}}}\left(L^{t_{1}t_{2}}\times \cos\theta_{k}\right)\;\;\\ {\vec{m}}_{j}^{t_{1}t_{2}}.y=\sum_{k=1}^{{\mathrm{StepNum}}^{t_{1}t_{2}}}\left(L^{t_{1}t_{2}}\times \sin\theta_{k}\right)\; \end{array}$$

where $\theta_{k}$ is the angle between the *x*-axis and the pedestrian’s heading direction measured by the geomagnetic sensor equipped by mobile devices and ${\mathrm{StepNum}}^{t_{1}t_{2}}$ is the number of measured steps during [$t_{1}$,$t_{2}$].

**Figure 5:**
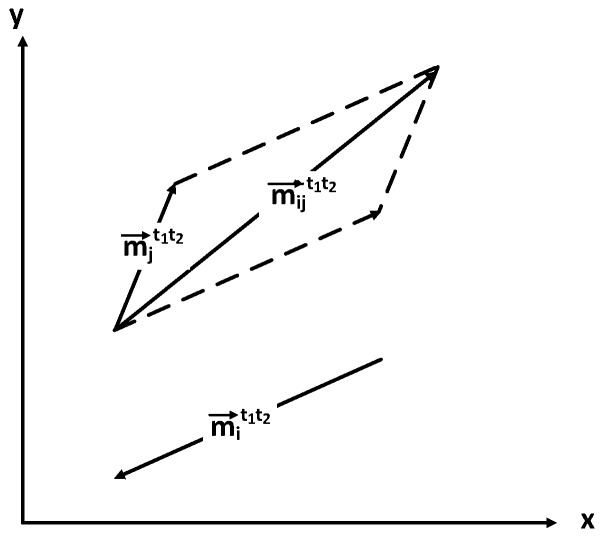
Relative movement of *j* towards *i*.

As shown by Figure 5, the relative movement of LIR *j* towards broadcaster *i* from $t_{1}$ to $t_{2}$, ${\vec{m}}_{ij}^{t_{1}t_{2}}$, can be calculated by:

(3) $$\begin{array}{c} {\vec{m}}_{ij}^{t_{1}t_{2}}={\vec{m}}_{i}^{t_{1}t_{2}}-{\vec{m}}_{j}^{t_{1}t_{2}}\;\\ {\vec{m}}_{ij}^{t_{1}t_{2}}.x={\vec{m}}_{i}^{t_{1}t_{2}}.x-{\vec{m}}_{j}^{t_{1}t_{2}}.x\;\\ {\vec{m}}_{ij}^{t_{1}t_{2}}.y={\vec{m}}_{i}^{t_{1}t_{2}}.y-{\vec{m}}_{j}^{t_{1}t_{2}}.y\; \end{array}$$

Let $d_{ij}^{t}$ be the distance between participants *i* and *j* at time *t* and $D^{t}$ = ${\left(d_{ij}^{t}\right)}_{M\times M}$ be the distance matrix at time *t*, such that:

(4) $$D^{t}=\left[\begin{array}{cccc} d_{11}^{t} & d_{12}^{t} & \cdots & d_{1M}^{t}\\ d_{21}^{t} & d_{22}^{t} & \cdots & d_{2M}^{t}\\ \vdots & \vdots & \ddots & \vdots \\ d_{M1}^{t} & d_{M2}^{t} & \cdots & d_{MM}^{t} \end{array}\right]\;\;\;\;\;$$

According to the free space radio propagation model [22], the relationship between $d_{ij}^{t}$ and $r_{ij}^{t}$ is denoted as:

(5) $$\begin{array}{c} \frac{d_{ij}^{t_{1}}}{d_{ij}^{t_{2}}}=10^{\frac{r_{ij}^{t_{2}}-r_{ij}^{t_{1}}}{10n}}\; \end{array}$$

where *n* is a measure of the influence of obstacles, like partitions, walls and doors.

Let $k_{t_{1}t_{2}}$ denote:

(6) $$k_{t_{1}t_{2}}=10^{\frac{r_{ij}^{t_{1}}-r_{ij}^{t_{2}}}{10n}}$$

Then, we have:

(7) $$\begin{array}{c} d_{ij}^{t_{2}}=k_{t_{1}t_{2}}\times d_{ij}^{t_{1}}\;\\ d_{ij}^{t_{3}}=k_{t_{1}t_{3}}\times d_{ij}^{t_{1}}\; \end{array}$$

**Figure 6:**
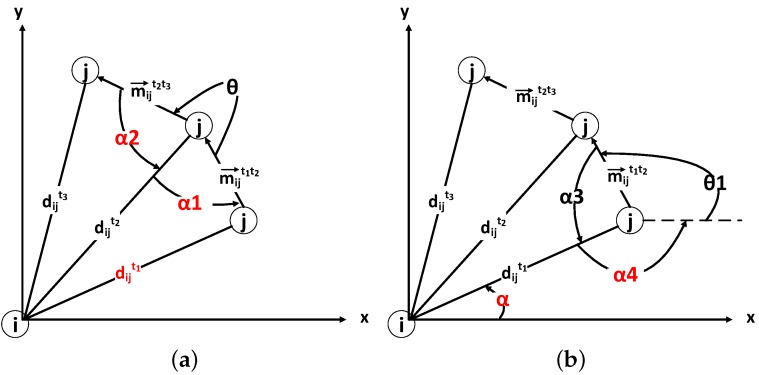
Targeted parameters: $d_{ij}^{t_{1}}$ (**a**) and *α* (**b**).

The problem is transformed as shown in Figure 6. Broadcaster *i*’s location at time $t_{1}$ is set to be the origin of the coordinates. The *y*-axis is pointing to the north, and the *x*-axis is pointing to the east. The direction of *j* towards *i* at $t_{1}$ is denoted by the angle *α* (see Figure 6b).

Let $\alpha_{1}$ and $\alpha_{2}$ denote the angle between $d_{ij}^{t_{1}}$ and ${\vec{m}}_{ij}^{t_{1}t_{2}}$ and the angle between $d_{ij}^{t_{2}}$ and ${\vec{m}}_{ij}^{t_{2}t_{3}}$ respectively, as shown in Figure 6a. According to the cosine theorem, $\alpha_{1}$ and $\alpha_{2}$ can be expressed by:

(8) $$\begin{array}{c} \cos\alpha_{1}=\frac{{d_{ij}^{t_{2}}}^{2}+{|{\vec{m}}_{ij}^{t_{1}t_{2}}|}^{2}-{d_{ij}^{t_{1}}}^{2}}{2d_{ij}^{t_{2}}|{\vec{m}}_{ij}^{t_{1}t_{2}}|}\;\\ \cos\alpha_{2}=\frac{{d_{ij}^{t_{2}}}^{2}+{|{\vec{m}}_{ij}^{t_{2}t_{3}}|}^{2}-{d_{ij}^{t_{3}}}^{2}}{2d_{ij}^{t_{2}}|{\vec{m}}_{ij}^{t_{2}t_{3}}|}\; \end{array}$$

(9) $$\begin{array}{c} \alpha_{1}=\arccos\frac{{d_{ij}^{t_{2}}}^{2}+{|{\vec{m}}_{ij}^{t_{1}t_{2}}|}^{2}-{d_{ij}^{t_{1}}}^{2}}{2d_{ij}^{t_{2}}|{\vec{m}}_{ij}^{t_{1}t_{2}}|}\;\\ \alpha_{2}=\arccos\frac{{d_{ij}^{t_{2}}}^{2}+{|{\vec{m}}_{ij}^{t_{2}t_{3}}|}^{2}-{d_{ij}^{t_{3}}}^{2}}{2d_{ij}^{t_{2}}|{\vec{m}}_{ij}^{t_{2}t_{3}}|}\; \end{array}$$

The angle *θ* between ${\vec{m}}_{ij}^{t_{1}t_{2}}$ and ${\vec{m}}_{ij}^{t_{2}t_{3}}$ can be calculated from ${\vec{m}}_{ij}^{t_{1}t_{2}}$ and ${\vec{m}}_{ij}^{t_{2}t_{3}}$. Besides, $\theta ,\alpha_{1},\alpha_{2}$ add up to 2π.

$$\alpha_{1}+\alpha_{2}+\theta =2\pi \;$$

Thus,

(10) $$\begin{array}{c} \arccos\frac{\left({k_{t_{1}t_{2}}}^{2}-1\right){d_{ij}^{t_{1}}}^{2}+{|{\vec{m}}_{ij}^{t_{1}t_{2}}|}^{2}}{2k_{t_{1}t_{2}}d_{ij}^{t_{1}}|{\vec{m}}_{ij}^{t_{1}t_{2}}|}+\arccos\frac{\left({k_{t_{1}t_{2}}}^{2}-{k_{t_{1}t_{3}}}^{2}\right){d_{ij}^{t_{1}}}^{2}+{|{\vec{m}}_{ij}^{t_{2}t_{3}}|}^{2}}{2k_{t_{1}t_{2}}d_{ij}^{t_{1}}|{\vec{m}}_{ij}^{t_{2}t_{3}}|}+\theta =2\pi \; \end{array}$$

By differentiation, the derivative of Equation (10) is always positive. Thus, the sufficient conditions for the monotonicity of Equation (10) are fulfilled. The transcendental Equation (10) can be solved by the binary search method.

From Figure 6b, we can easily see that $\alpha +\alpha_{4}=\pi$. The angle between ${\vec{m}}_{ij}^{t_{1}t_{2}}$ and $d_{ij}^{t_{1}}$, denoted by $\alpha_{3}$, can be calculated by:

(11) $$\begin{array}{c} \alpha_{3}=\arccos\frac{{d_{ij}^{t_{1}}}^{2}+{|{\vec{m}}_{ij}^{t_{1}t_{2}}|}^{2}-{d_{ij}^{t_{2}}}^{2}}{2d_{ij}^{t_{1}}|{\vec{m}}_{ij}^{t_{1}t_{2}}|}\; \end{array}$$

Besides, $\theta_{1},\alpha_{3},\alpha_{4}$ add up to 2π.

$$\alpha_{3}+\alpha_{4}+\theta_{1}=2\pi \;$$

Thus,

(12) $$\begin{array}{c} \alpha =\alpha_{3}+\theta_{1}-\pi =\arccos\frac{{d_{ij}^{t_{1}}}^{2}+{|{\vec{m}}_{ij}^{t_{1}t_{2}}|}^{2}-{k_{t_{1}t_{2}}^{2}\times d_{ij}^{t_{1}}}^{2}}{2d_{ij}^{t_{1}}|{\vec{m}}_{ij}^{t_{1}t_{2}}|}+\theta_{1}-\pi \; \end{array}$$

From the above, LIR *j* can calculate its relative location to broadcaster *i* at $t_{1}$. Given the location broadcast from *i*, LIR *j* can calculate its location at $t_{1}$. From Equation (2), LIR *j* knows ${\vec{m}}_{j}^{t_{1}t_{2}}$ and ${\vec{m}}_{j}^{t_{2}t_{3}}$; thus, its location at $t_{2}$ and $t_{3}$ can be calculated.

Table 2, which is our preliminary results in [19], shows the localization accuracy considering different distances between the broadcasters and the LIRs. When the localization accuracy requirement *r* is higher, it requires a shorter distance $a_{r}$ between the broadcasters and the LIRs. Note that the communication range of WiFi is denoted by *λ*. When *r* is greater than 25 m, $a_{r}$ is equal to *λ* meters.

### 4.2. Energy Consumption Model

Let $e_{g}$ and $e_{c}$ be the power of GPS and the cellular network for localization, respectively. Let $e_{w1}$ and $e_{w2}$ be the power of WiFi communication for sending and receiving data, where $e_{g}>e_{c}>e_{w1}>e_{w2}$. Then, we will study the power of mobile participants with different roles in collaborative localization.

**Figure 7:**
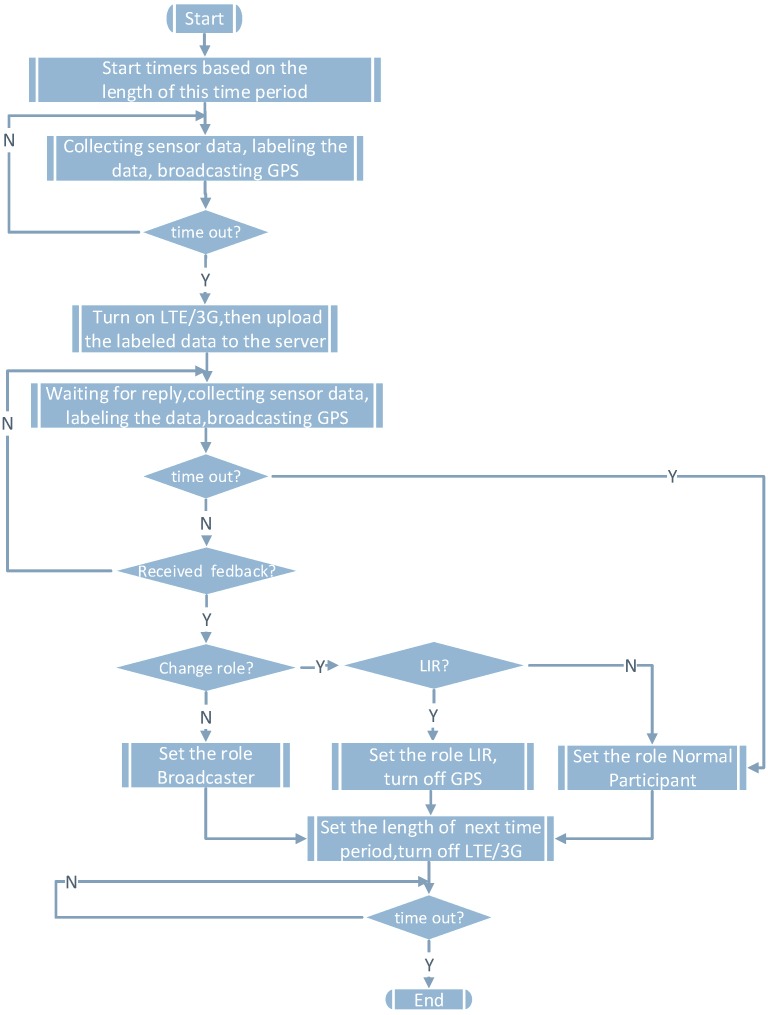
The broadcaster’s function flow.

**Figure 8:**
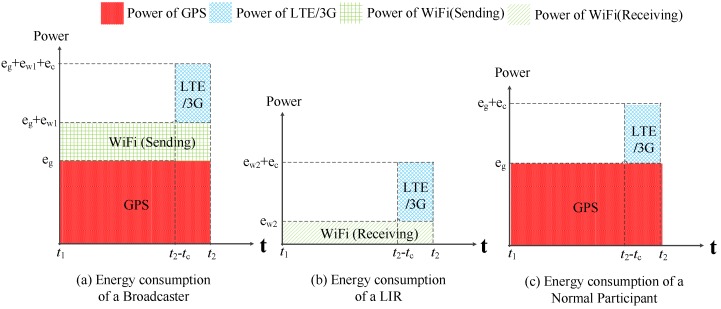
Energy consumption of different roles of a participant during [$t_{1},t_{2}$]. (**a**) Energy consumption of a Broadcaster; (**b**) Energy consumption of a LIR; (**c**) Energy consumption of a Normal Participant.

Figure 7 shows the function flow of the broadcaster. During [$t_{1},t_{2}-t_{c}$], the broadcaster uses GPS to obtain its location and uses WiFi to broadcast its location to the surrounding LIRs. During [$t_{2}-t_{c}$,$t_{2}$], the broadcaster uses the cellular network to report sensor data. We can easily model the energy consumption of the broadcaster during [$t_{1},t_{2}$] in Figure 8a. Similarly, we can illustrate the energy consumption of an LIR and a normal participant in Figure 8b,c.

Let $e_{b}^{t_{1}t_{2}}$ be the power of a broadcaster for collaboration localization in [$t_{1},t_{2}$], where ∀$t_{1}$,$t_{2}$∈T.

(13) $$e_{b}^{t_{1}t_{2}}=\left(\left(e_{g}+e_{w1}\right)\times \left(t_{2}-t_{1}\right)+e_{c}\times t_{c}\right)/\left(t_{2}-t_{1}\right)\;$$

Let $e_{l}^{t_{1}t_{2}}$ be the power of an LIR during [$t_{1},t_{2}$].

(14) $$e_{l}^{t_{1}t_{2}}=\left(e_{w2}\times \left(t_{2}-t_{1}\right)+e_{c}\times t_{c}\right)/\left(t_{2}-t_{1}\right)\;$$

Let $e_{n}^{t_{1}t_{2}}$ be the power of a normal participant during [$t_{1},t_{2}$].

(15) $$e_{n}^{t_{1}t_{2}}=\left(e_{g}\times \left(t_{2}-t_{1}\right)+e_{c}\times t_{c}\right)/\left(t_{2}-t_{1}\right)\;$$

We use Boolean $b_{m}^{t_{1}t_{2}}$ to indicate whether a mobile participant *m* is a broadcaster during [$t_{1},t_{2}$]. Similarly, we use Boolean $n_{m}^{t_{1}t_{2}}$ to indicate whether a mobile participant *m* is a normal participant during $\left[t_{1},t_{2}\right]$.

Let $p_{ij}^{t_{1}t_{2}}$ be the physical connectivity between participant *i* and *j* during $\left[t_{1},t_{2}\right]$. Let $P^{t_{1}t_{2}}$ = ${\left(p_{ij}^{t_{1}t_{2}}\right)}_{M\times M}$ be the physical connectivity matrix during $\left[t_{1},t_{2}\right]$ , where:

(16) $$P^{t_{1}t_{2}}=\left[\begin{array}{cccc} p_{11}^{t_{1}t_{2}} & p_{12}^{t_{1}t_{2}} & \cdots & p_{1M}^{t_{1}t_{2}}\\ p_{21}^{t_{1}t_{2}} & p_{22}^{t_{1}t_{2}} & \cdots & p_{2M}^{t_{1}t_{2}}\\ \vdots & \vdots & \ddots & \vdots \\ p_{M1}^{t_{1}t_{2}} & p_{M2}^{t_{1}t_{2}} & \cdots & p_{MM}^{t_{1}t_{2}} \end{array}\right]\;\;\;\;\;$$

The power of all of the participants during $\left[t_{1},t_{2}\right]$, $E^{t_{1}t_{2}}$, consists of three parts: the power of all of the broadcasters during this period ($E_{b}^{t_{1}t_{2}}$), the power of the LIRs ($E_{l}^{t_{1}t_{2}}$) and the power of the normal participants ($E_{n}^{t_{1}t_{2}}$). We calculate these three parts in the following.

Given $|B^{t_{1}t_{2}}|$ and $e_{b}^{t_{1}t_{2}}$, the power of all of the data broadcasters during this period can be calculated by:

(17) $$\begin{array}{cc} E_{b}^{t_{1}t_{2}} & =|B^{t_{1}t_{2}}|e_{b}^{t_{1}t_{2}}\\ & =\sum_{m=1}^{\left|M\right|}b_{m}^{t_{1}t_{2}}e_{b}^{t_{1}t_{2}}\; \end{array}$$

According to $B^{t_{1}t_{2}}$ and $P^{t_{1}t_{2}}$, we can get $N^{t_{1}t_{2}}$. Thus, the power of all of the normal participants during this period can be calculated by:

(18) $$\begin{array}{cc} E_{n}^{t_{1}t_{2}} & =|N^{t_{1}t_{2}}|e_{n}^{t_{1}t_{2}}\\ & =\sum_{m=1}^{\left|M\right|}n_{m}^{t_{1}t_{2}}e_{n}^{t_{1}t_{2}}\; \end{array}$$

For the remaining LIRs, their power during this period can be calculated by:

(19) $$E_{l}^{t_{1}t_{2}}=\left(|M\right|-\sum_{m=1}^{\left|M\right|}b_{m}^{t_{1}t_{2}}-\sum_{m=1}^{\left|M\right|}n_{m}^{t_{1}t_{2}})\times e_{l}^{t_{1}t_{2}}\;$$

Finally, the power of all of the participants during this collaborative localization time period, $E^{t_{1}t_{2}}$, can be calculated by:

(20) $$\begin{array}{cc} E^{t_{1}t_{2}} & =E_{b}^{t_{1}t_{2}}+E_{l}^{t_{1}t_{2}}+E_{n}^{t_{1}t_{2}}\\ & =\sum_{m=1}^{\left|M\right|}b_{m}^{t_{1}t_{2}}e_{b}^{t_{1}t_{2}}+\left(|M\right|-\sum_{m=1}^{\left|M\right|}b_{m}^{t_{1}t_{2}}-\sum_{m=1}^{\left|M\right|}n_{m}^{t_{1}t_{2}})\times e_{l}^{t_{1}t_{2}}+\sum_{m=1}^{\left|M\right|}n_{m}^{t_{1}t_{2}}e_{n}^{t_{1}t_{2}}\; \end{array}$$

### 4.3. Problem Formulation

The main goal of this work is to find an optimal broadcaster set $B^{t_{1}t_{2}}$ and the length of each collaborative localization period, so that the power of all of the participants is minimized. To prevent some broadcasters from possibly consuming too much energy, our model considers the current remaining battery of participants, which is denoted by $c_{m}^{t}$, ∀m∈M,t∈T. We formulate the BSSP in the following:

Minimize: $\sum_{m=1}^{\left|M\right|}b_{m}^{t_{1}t_{2}}e_{b}^{t_{1}t_{2}}+\left(|M\right|-\sum_{m=1}^{\left|M\right|}b_{m}^{t_{1}t_{2}}-\sum_{m=1}^{\left|M\right|}n_{m}^{t_{1}t_{2}})\times e_{l}^{t_{1}t_{2}}+\sum_{m=1}^{\left|M\right|}n_{m}^{t_{1}t_{2}}e_{n}^{t_{1}t_{2}}$

Subject to:

(21) $$b_{m}^{t_{1}t_{2}}=\left\{0,1\right\},\forall m\in M,t_{2}\in T\wedge t_{2}>t_{1}\;\;\;\;\;\;\;\;\;\;\;\;\;\;\;\;\;\;\;\;\;\;\;\;\;\;\;\;\;\;\;\;\;\;\;\;\;\;\;\;\;\;\;\;$$

(22) $$e_{b}^{t_{1}t_{2}}=\left(\left(e_{g}+e_{w1}\right)\times \left(t_{2}-t_{1}\right)+e_{c}\times t_{c}\right)/\left(t_{2}-t_{1}\right),t_{2}\in T\wedge t_{2}>t_{1}\;\;\;\;\;\;\;$$

(23) $$e_{l}^{t_{1}t_{2}}=\left(e_{w2}\times \left(t_{2}-t_{1}\right)+e_{c}\times t_{c}\right)/\left(t_{2}-t_{1}\right),t_{2}\in T\wedge t_{2}>t_{1}\;\;\;\;\;\;\;\;\;\;\;\;\;\;\;\;\;\;\;\;$$

(24) $$e_{n}^{t_{1}t_{2}}=\left(e_{g}\times \left(t_{2}-t_{1}\right)+e_{c}\times t_{c}\right)/\left(t_{2}-t_{1}\right),t_{2}\in T\wedge t_{2}>t_{1}\;\;\;\;\;\;\;\;\;\;\;\;\;\;\;\;\;\;\;\;$$

(25) $$c_{m}^{t_{1}}\geq \mu ,\forall m\in B^{t_{1}t_{2}},t_{2}\in T\wedge t_{2}>t_{1}\;\;\;\;\;\;\;\;\;\;\;\;\;\;\;\;\;\;\;\;\;\;\;\;\;\;\;\;\;\;\;\;\;\;\;\;\;\;\;\;\;\;\;\;\;\;\;\;\;\;\;\;\;$$

(26) $$p_{ij}^{t_{1}t_{2}}=\left\{\begin{array}{cc} & 0\;,\;d_{ij}^{t_{1}}>a_{r}-v\times \left(t_{2}-t_{1}\right),\forall i,j\in M,t_{2}\in T\wedge t_{2}>t_{1}\\ & 1\;,\;d_{ij}^{t_{1}}\leq a_{r}-v\times \left(t_{2}-t_{1}\right),\forall i,j\in M,t_{2}\in T\wedge t_{2}>t_{1} \end{array}\right.\;$$

(27) $$n_{m}^{t_{1}t_{2}}=\left\{\begin{array}{cc} & 0\;,\;\left(\sum_{j=1}^{\left|M\right|}p_{mj}^{t_{1}t_{2}}\geq h,\forall m\in M\setminus B^{t_{1}t_{2}},t_{2}\in T\wedge t_{2}>t_{1}\right)\vee \left(\forall m\in B^{t_{1}t_{2}}\right)\\ & 1\;,\;\sum_{j=1}^{\left|M\right|}p_{mj}^{t_{1}t_{2}}<h,\forall m\in M\setminus B^{t_{1}t_{2}},t_{2}\in T\wedge t_{2}>t_{1} \end{array}\right.\;$$

Equation (21) is the integer constraint. Equations (22)–(24) calculate the power of the participants in the three different roles during $\left[t_{1},t_{2}\right]$. Equation (25) enforces that the remaining battery of each broadcaster must be above *μ*. Equation (26) enforces that the physical connectivity matrix of this period is determined by the current distance matrix, the length of this period, the location accuracy requirement of the task and the moving speed of pedestrian *v*. Equation (27) enforces that each LIR has to receive broadcasts from at least *h* broadcasters so that it can calculate its location. In our proposed device to device localization method, h=1. It is worth noting that, simply by changing $a_{r}$ and *h*, other collaborative localization methods can also be represented by our proposed model.

## 5. Our Proposed Solutions

We propose two heuristic algorithms to solve the BSSP. The first one is a greedy-based broadcaster selection (GBS) algorithm. Algorithm 1 shows the pseudocode of the GBS algorithm. Based on the current distance matrix of all of the participants, the localization accuracy of the task and the moving speed of the pedestrian, GBS is able to get the approximate optimal set of broadcasters of the following collaborative localization period and the length of the following period.

Lines 9–29 show the inner while loop where the length of the period is fixed; thus, the solution is a local solution. We denote the power of participants in the local solution by $E_{local}$. In the beginning, $B^{t_{1}t_{2}}$ is empty, and $N^{t_{1}t_{2}}$ equals M. In each round of the while loop, the participant that saves the most energy will be selected as a new broadcaster during this fixed time period. The selection of broadcasters will continue until the power of all participants during this period, $E^{t_{1}t_{2}}$, cannot be further improved. After the while loop, we can get a local optimal broadcasters’ set under a given time $t_{2}$. After the outer for loop (Lines 3–34) we can get the global optimal broadcasters’ set and its corresponding length of the period. By using $B^{t_{1}t_{2}}$, we can easily get the set of normal participants $N^{t_{1}t_{2}}$. The remaining participants, $M\setminus (B^{t_{1}t_{2}}\cup N^{t_{1}t_{2}})$, are LIRs. All of the roles of the participants of the next period and the length of the next period are then calculated.

We also propose a simulated annealing (SA)-based [23] broadcaster selection (SABS) algorithm. SA is a probabilistic algorithm that makes a good approximation to the global optimal solution of the optimization problem in a large search space.

The aim of the SABS is to get an approximate optimal set of broadcasters for the following collaborative localization period and the length of that period. The SABS algorithm includes a sub-algorithm called SubSABS.

Algorithm 2 shows the pseudocode of the SubSABS algorithm. SubSABS can find an approximate optimal $E^{t_{1}t_{2}}$ only when the length of this period $|t_{2}-t_{1}|$ and the number of the broadcasters $|B^{t_{1}t_{2}}|$ are given. Since the length of the period and the number of broadcasters during this period are fixed, the solution is a local solution. We denote the power of participants and the set of broadcasters in the local solution by $E_{local}$ and $B_{local}$. Lines 1–7 are the initialization phase. Initially, it generates a feasible $B^{t_{1}t_{2}}$ as the starting point. In each iteration of the outer while loop (Lines 8–30), a neighbor set of $B^{t_{1}t_{2}}$ is generated, which is denoted by $B_{next}$. If $B_{local}^{next}$ can save more energy than $B_{local}$, then we accept $B_{local}^{next}$ as a new set of broadcasters. Otherwise, we accept $B_{local}^{next}$ based on the probability of avoiding falling into a local minimum. The probability of acceptance is an exponentially decreasing function with parameter $exp(-\Delta energy/T^{\circ })$, where $T^{\circ }$ is the current temperature. After each iteration, the probability of acceptance decreases. It can compute the suboptimal $E_{local}$ given specific $|t_{2}-t_{1}|$ and $|B^{t_{1}t_{2}}|$. This result will be used in the SABS algorithm.


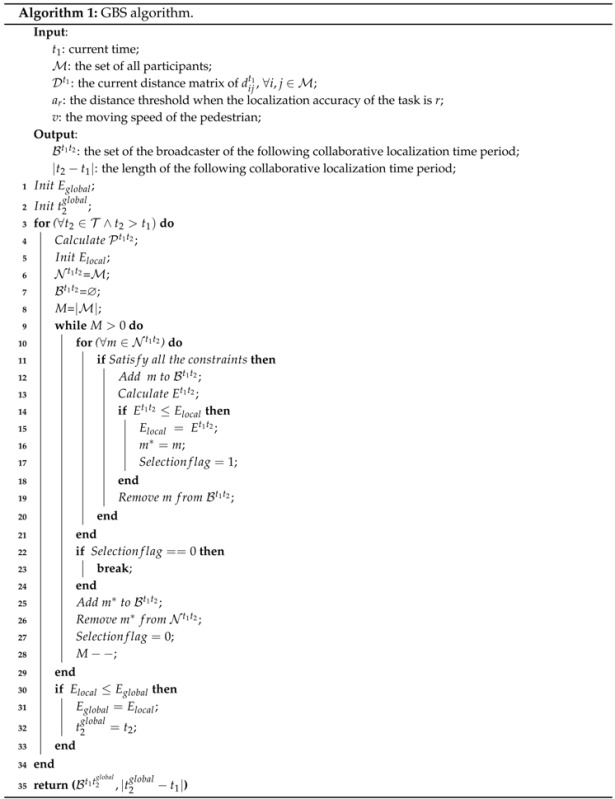



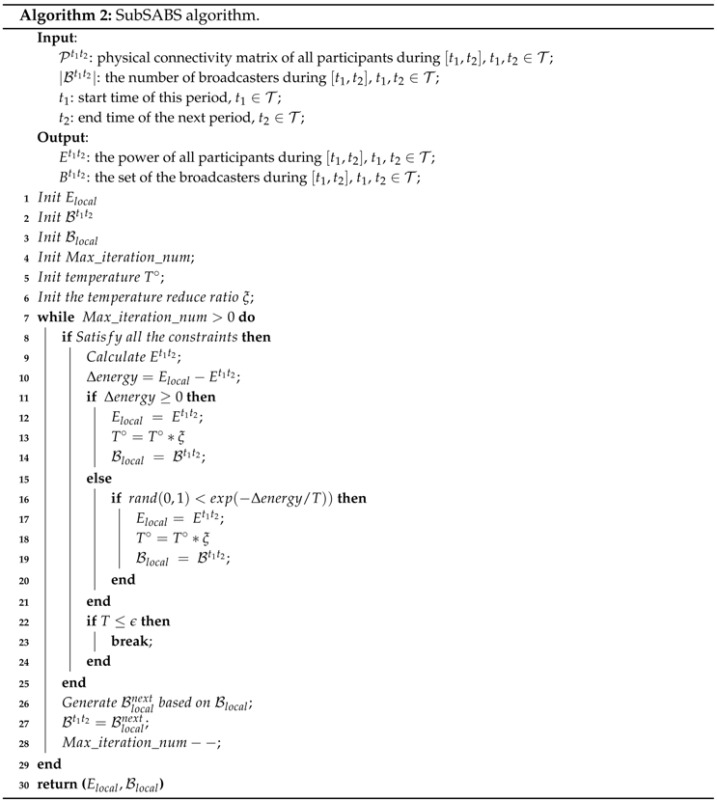


Based on SubSABS, SABS can easily find the approximate optimal set of broadcasters for the following collaborative localization period and the length of that period. Algorithm 3 shows the pseudocode of the SABS algorithm. It can find an approximate solution by varying the number of broadcasters $|B^{t_{1}t_{2}}|$ and calling the SubSABS algorithm. Even though the optimal $|B^{t_{1}t_{2}}|$ is not known in advance, the SABS algorithm can find the approximate solution $E_{local}$ by calling the SubSABS algorithm $O(log_{2}^{\left|M\right|})$ times. After the outer for loop, SABS algorithm can find the approximate length of the next period $|t_{2}^{glocal}-t_{1}|$ and the set of broadcasters during this period $B^{t_{1}t_{2}^{glocal}}$. Similar to the GBS algorithm, based on $B^{t_{1}t_{2}^{glocal}}$, the roles of all participants in the next period can be calculated.


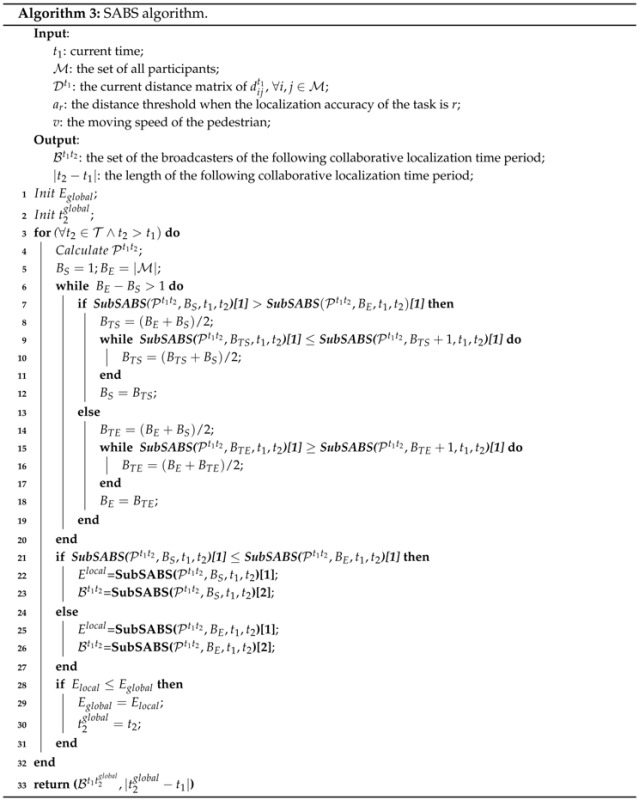


## 6. Performance Evaluation

We evaluate the performance of the proposed collaborative strategy using the GeoLife (Microsoft Research Asia) dataset [24], where real movement traces of ordinary citizens are used to represent mobile users in the considered scenario. The GeoLife project of Microsoft Research Asia has collected 182 volunteers’ (ordinary citizens) trajectories in Beijing from April 2007–August 2012. A GPS trajectory of this dataset is represented by a sequence of time-stamped points, each of which contains the information of latitude, longitude and altitude.

**Figure 9:**
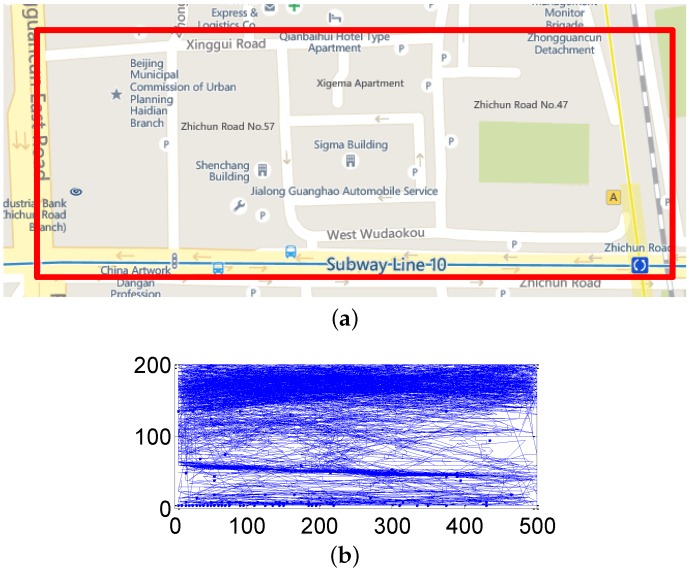
Simulation setup: (**a**) Simulation region (red rectangle); (**b**) User trajectories.

We store all of the trajectories in a geographical MySQL database and find a 200 × 500 m${}^{2}$ region that is of high movement density, as shown in Figure 9a.

There are 612 trajectories in this region, as shown in Figure 9b. All 612 trajectories in the region are taken as mobile participants, *i.e.*, |M| = 612. Since a sensing task usually lasts for some time, some trajectories are too short to experiment. Thus, we choose the trajectories with a length longer than 20. After screening, there are 178 trajectories in total, *i.e.*, |M| = 178. We set the parameters in our energy model according to [25,26,27], where $e_{g}$ = 355 mW, $e_{c}$ = 268 mW, $e_{w1}$ = 240 mW and $e_{w2}$ = 50 mW. As the GeoLife dataset does not provide battery information, we set *μ* = 0%, which means that every participant has the possibility to be a broadcaster.

**Figure 10:**
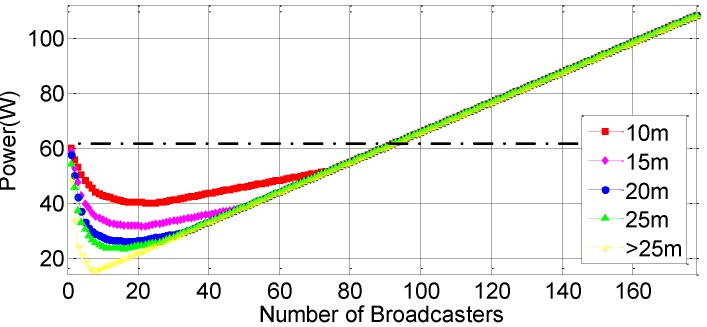
The impact of the number of broadcasters.

First, we evaluate how the number of broadcasters will influence the total power under different localization accuracy requirements. In this experiment, $l/t_{c}$ is set to 20. Figure 10 shows the power of all participants varying the number of broadcasters. Initially, the power decreases with the number of broadcasters. However, after reaching the optimal points, the power increases with the number of broadcasters. This is because excessive broadcasters consume energy for GPS localization and broadcasting their locations. The black dotted line in this figure shows the power of all participants without collaborative localization. We can see that if the broadcasters reach a certain number, collaborative localization does not save more energy than GPS localization.

**Figure 11:**
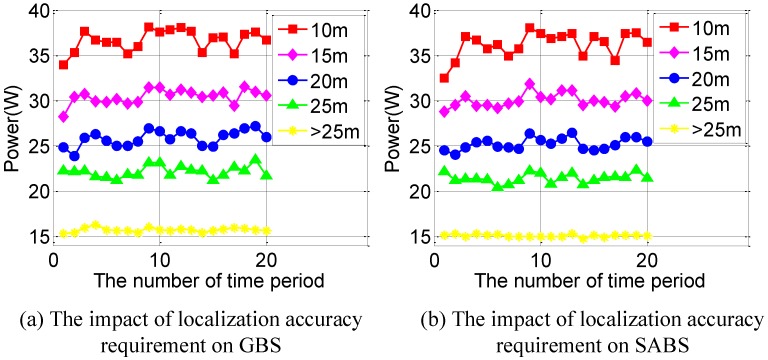
The impact of the localization requirement on our method. (**a**) The impact of the localization accuracy requirement on GBS; (**b**) The impact of the localization accuracy requirement on SABS.

Next, we evaluate our proposed solution under different localization accuracy requirements. Figure 11a,b shows the impact of localization accuracy on GBS and SABS. As the localization accuracy requirement increases, both the GBS and the SABS consume more energy. This is because more broadcasters are needed to satisfy the accuracy requirement. It is worth noting that GBS performs better than SABS in some cases. This is because the number of iterations of SubSABS is set to 5000. If we increase the number of iterations, SABS performs better than GBS in all cases.

**Figure 12:**
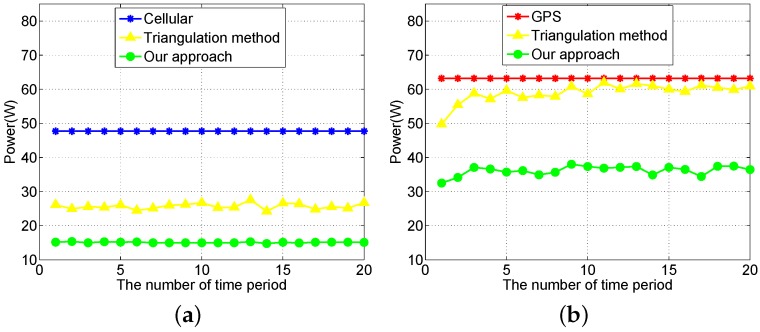
Comparison with the existing approach. (**a**) Low localization accuracy requirement; (**b**) High localization accuracy requirement.

**Figure 13:**
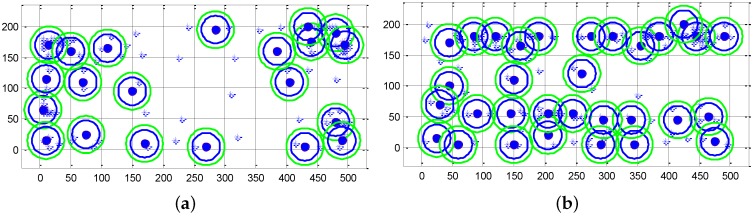
Time slice with maximum and minimum energy saving. (**a**) Time slice with maximum energy saving; (**b**) Time slice with minimum energy saving.

Then, we compare the power consumption of our approach to the existing solutions. We compare the standard triangulation method for collaborative localization with our proposed device to device localization method. When we use the triangulation method for collaborative localization, each LIR relies on three broadcasters’ locations to calculate its own location. Figure 12a compares our approach to the cellular and triangulation method when the localization accuracy requirement is greater than 25 m. Our approach can save 68% and 41% energy on average compared to the cellular and triangulation localization methods, respectively. Figure 12b compares the power consumption of our approach to the GPS sampling and triangulation method when the localization accuracy requirement is 10 m. In the traditional approach, all mobile participants turn on their GPS to perform localization periodically [11,28]. Our approach can save 43% and 38% of the energy on average compared to GPS and triangulation localization, respectively. In the best case, our approach can save 48% of the energy compared to GPS localization. In the worst case, our approach can save 40% of the energy compared to GPS localization. Figure 13a shows the time slice of user locations with maximum energy saving (the best case), while Figure 13b shows the time slice with minimum energy saving (the worst case). The radius of the outer green circles in the figure is equal to $a_{r}$, which is the distance threshold corresponding to the localization accuracy requirement. The radius of the inner blue circles is equal to $a_{r}-v*l$, so that all of the LIRs within the blue circle will not move out of the green circle during a collaborative localization period, where *v* is the moving speed of the pedestrian and *l* is the length of the collaborative localization period.

**Figure 14:**
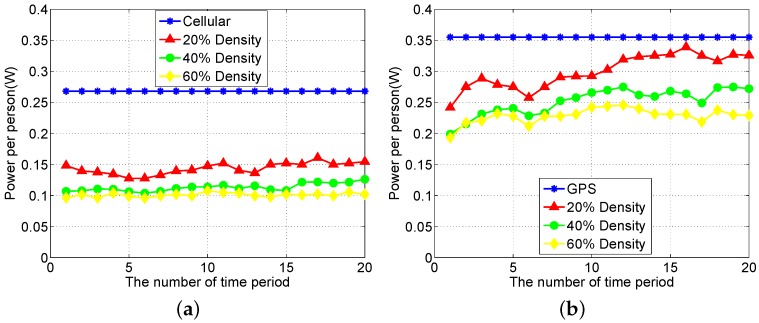
The influence of participants’ density. (**a**) The influence of participants’ density under the low localization accuracy requirement; (**b**) The influence of participants’ density under the high localization accuracy requirement.

Furthermore, we study how the participant density will influence our proposed method. We choose 20%, 40% and 60% of the total participants to do the experiment, respectively. We choose the subsets of participants so that others can repeat this experiment. As the number of participants varies under different participant density, we evaluate the average power per participant during each time period. Figure 14a shows the influence of participant density when the localization accuracy is greater than 25 m, while Figure 14b shows the influence of participant density when the localization accuracy is 10 m. From Figure 14, we can see that as the participant density increases, our method proposed performs better under both localization accuracy requirements.

Finally, we evaluate the performance of our proposed algorithm. In a specific case when $e_{n}^{t_{1},t_{2}}$ is set to $e_{b}^{t_{1},t_{2}}$ and the length of the time period is fixed, the BSSP can be converted to the well-known set cover problem (SCP), ∀$t_{1},t_{2}$∈T. Then, we can get the optimal solution on the specific case using CPLEX 12.6 [29]. In our experiment, the localization accuracy is set to 25 m, and $l/t_{c}$ is set to 20. We choose the time slice with the most participants; then, we filter the participants who are at the same locations. Under this condition, there are 325 participants in total, *i.e*., |*M*| = 325.

**Figure 15:**
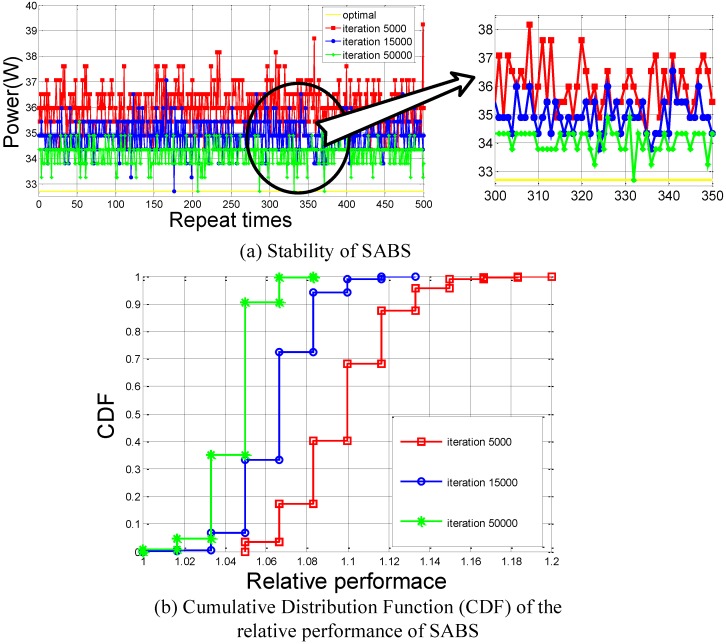
The performance of our proposed solution. (**a**) Stability of SABS; (**b**) Cumulative Dirstrbution Function (CDF) of the relative performance of SABS.

Figure 15a shows the stability of the SABS algorithm with different numbers of iterations. When the number of iterations is set to 5000, SABS is 90.2% to the optimal on average. When the number of iterations is set to 15,000, SABS is 93.4% to the optimal on average and has a probability of 0.2% to reach the optimal. When the number of iterations is set to 50,000, SABS is as close as 95.5% to the optimal on average. It has a probability of 0.8% to reach the optimal. Figure 15b shows the cumulative distribution function (CDF) of the relative performance of the SABS algorithm with different numbers of iterations. As the number of iterations increases, SABS performs better. Even when the number of iterations is set to only 5000, our proposed solution is already much better than the existing solutions, as shown in Figure 12.

In summary, our solution enables flexible adjustment on the localization accuracy according to the application requirement and minimizes the energy consumption for localization. Under all localization accuracy requirements, our approach performs better than the existing approaches.

## 7. Conclusions and Future Work

In this paper, we propose a collaborative outdoor localization architecture, which aims to reduce the total energy consumption of mobile participants during the location information collection process. This architecture is capable of flexible adjustment on the localization accuracy according to the application requirement. A mathematical model is set up to measure the total energy consumption of mobile participants. We formulate the BSSP and propose two novel algorithms, namely SABS and GBS algorithm, to minimize the energy consumption of all participants by coordinating between the broadcasters and the LIRs. The performances of the two algorithms are evaluated based on extensive simulations using real mobility traces. Simulation results show that our proposed localization strategy can save up to 68% of the total energy and achieve high localization accuracy.

For future work, vehicle participants could be taken into account to enhance the stability of the system and to further reduce the energy consumption.

## Figures and Tables

**Table 1:** List of notations.

*Notation*	*Explanation*
M	The set of all participants.
T	The set of time T= $\left\{t_{s},t_{s}+1,...,t_{e}\right\}$, where $t_{s}$ and $t_{e}$ are the start time and end time of a task.
$B^{t_{1}t_{2}}$	The set of broadcasters during $\left[t_{1},t_{2}\right]$,$\forall t_{1},t_{2}$ ∈ T.
$N^{t_{1}t_{2}}$	The set of normal participants during $\left[t_{1},t_{2}\right]$,$\forall t_{1},t_{2}$ ∈ T.
*l*	The length of the following collaborative localization period.
$t_{c}$	The time that the participants need for sending data and receiving roles.
$r_{ij}^{t}$	The RSSI between *i* to *j* at time *t*, ∀i,j ∈ M, ∀t ∈ T.
${\vec{m}}_{i}^{t_{1}t_{2}}$	The movement of participant *i* from $t_{1}$ to $t_{2}$, ∀i ∈ M, $\forall t_{1},t_{2}$ ∈ T.
${\vec{m}}_{ij}^{t_{1}t_{2}}$	The relative movement between *i* and *j* from $t_{1}$ to $t_{2}$, ∀i,j ∈ M, $\forall t_{1},t_{2}$ ∈ T.
$L^{t_{1}t_{2}}$	The step length of pedestrians during [$t_{1}$,$t_{2}$], $\forall t_{1},t_{2}$ ∈ T.
$A_{max}^{t_{1}t_{2}}$	The maximum acceleration during [$t_{1}$,$t_{2}$], $\forall t_{1},t_{2}$ ∈ T.
$A_{min}^{t_{1}t_{2}}$	The minimum acceleration during [$t_{1}$,$t_{2}$], $\forall t_{1},t_{2}$ ∈ T.
$d_{ij}^{t}$	The distance between participant *i* and participant *j* at time t, ∀i, *j* ∈ M, ∀t ∈ T.
$D^{t}$	The distance Matrix of $d_{ij}^{t}$, ∀i,j ∈ M, ∀t ∈ T.
$a_{r}$	The distance threshold for a given localization accuracy requirement *r*.
*λ*	The communication range of WiFi.
$e_{g}$	Power of the GPS.
$e_{c}$	Power of cellular the network.
$e_{w1}$	Power of WiFi when sending.
$e_{w2}$	Power of WiFi when receiving.
$b_{m}^{t_{1}t_{2}}$	Boolean to indicate whether *m* is a broadcaster during $\left[t_{1},t_{2}\right]$, ∀m∈M, $\forall t_{1},t_{2}$ ∈ T.
$n_{m}^{t_{1}t_{2}}$	Boolean to indicate whether *m* is a normal participant during $\left[t_{1},t_{2}\right]$, ∀m∈M $\forall t_{1},t_{2}$ ∈ T.
$e_{b}^{t_{1}t_{2}}$	Power of a broadcaster during $\left[t_{1},t_{2}\right]$, $\forall t_{1},t_{2}$∈ T.
$e_{l}^{t_{1}t_{2}}$	Power of an LIRduring $\left[t_{1},t_{2}\right]$, $\forall t_{1},t_{2}$∈ T.
$e_{n}^{t_{1}t_{2}}$	Power of a normal participant during $\left[t_{1},t_{2}\right]$, $\forall t_{1},t_{2}$∈ T.
$p_{ij}^{t_{1}t_{2}}$	The physical connectivity between participant *i* and *j* during $\left[t_{1},t_{2}\right]$, ∀i,j ∈ M, $\forall t_{1},t_{2}$ ∈ T.
$P^{t_{1}t_{2}}$	The physical connectivity matrix of $p_{ij}^{t_{1}t_{2}}$, ∀i,j ∈ M, $\forall t_{1},t_{2}$ ∈ T.
$E^{t_{1}t_{2}}$	Power of all participants during $\left[t_{1},t_{2}\right]$, $\forall t_{1},t_{2}$∈ T.
$E_{b}^{t_{1}t_{2}}$	Power of all broadcasters during $\left[t_{1},t_{2}\right]$, $\forall t_{1},t_{2}$∈ T.
$E_{l}^{t_{1}t_{2}}$	Power of all LIR during $\left[t_{1},t_{2}\right]$, $\forall t_{1},t_{2}$∈ T.
$E_{n}^{t_{1}t_{2}}$	Power of all normal participants during $\left[t_{1},t_{2}\right]$, $\forall t_{1},t_{2}$∈ T.
*μ*	The required remaining battery level of each broadcaster.

**Table 2:** Localization accuracy.

Localization accuracy requirement (*r*)	10 m	15 m	20 m	25 m	>25 m
Distance threshold between devices ($a_{r}$)	30 m	40 m	50 m	60 m	*λ* m

## References

[1] Zhao Q., Zhu Y., Zhu H., Cao J., Xue G., Li B. Fair energy-efficient sensing task allocation in participatory sensing with smartphones. Proceedings of the IEEE International Conference on Computer Communications (INFOCOM).

[2] Sheng X., Tang J., Zhang W. Energy-efficient collaborative sensing with mobile phones. Proceedings of the IEEE International Conference on Computer Communications (INFOCOM).

[3] König I., Memon A.Q., David K. Energy consumption of the sensors of Smartphones. Proceedings of the VDE International Symposium on Wireless Communication Systems (ISWCS).

[4] Xi T., Edith N., Song Z., Tian Y., Gong X., Wang W. Energy-Efficient Collaborative Localization for Participatory Sensing System. Proceedings of the IEEE Global Communications Conference (Globecom).

[5] Macias E., Suarez A., Lloret J. (2013). Mobile sensing systems. Sensors.

[6] Dutta P., Aoki P.M., Kumar N., Mainwaring A., Myers C., Willett W., Woodruff A. Common sense: Participatory urban sensing using a network of handheld air quality monitors. Proceedings of the ACM Conference on Embedded Networked Sensor Systems (SenSys).

[7] Kanjo E. (2010). NoiseSPY: A Real-Time Mobile Phone Platform for Urban Noise Monitoring and Mapping. Mob. Netw. Appl..

[8] Zhang Z., Liu T., Chen D., Zhang W. (2014). Novel Algorithm for Identifying and Fusing Conflicting Data in Wireless Sensor Networks. Sensors.

[9] Tuncay G.S., Benincasa G., Helmy A. (2013). Autonomous and distributed recruitment and data collection framework for opportunistic sensing. Mob. Comput. Commun. Rev..

[10] Reddy S., Estrin D., Srivastava M. (2010). Recruitment framework for participatory sensing data collections. IEEE Pervasive Comput..

[11] Song Z., Zhang B., Liu C.H., Vasilakos A.V., Ma J., Wang W. QoI-Aware Energy-Efficient Participant Selection. Proceedings of the IEEE Communications Society Conference on Sensor and Ad Hoc Communications and Networks (SECON).

[12] Lu H., Lane N.D., Eisenman S.B., Campbell A.T. (2010). Bubble-sensing: Binding sensing tasks to the physical world. Pervasive Mob. Comput..

[13] Lu H., Yang J., Liu Z., Lane N.D., Choudhury T., Campbell A.T. The jigsaw continuous sensing engine for mobile phone applications. Proceedings of the ACM Conference on Embedded Networked Sensor Systems (SenSys).

[14] Shafer I., Chang M.L. Movement detection for power-efficient smartphone WLAN localization. Proceedings of the ACM International Conference on Modeling, Analysis, and Simulation of Wireless and Mobile Systems (MSWiM ).

[15] Baier P., Durr F., Rothermel K. PSense: Reducing Energy Consumption in Public Sensing Systems. Proceedings of the IEEE International Conference on Advanced Information Networking and Applications (AINA).

[16] Johnson T.A., Seeling P. Localization using bluetooth device names. Proceedings of the ACM/IEEE International Symposium on Mobile Ad Hoc Networking and Computing (MobiHoc).

[17] Xiao B., Chen H., Zhou S. (2008). Distributed localization using a moving beacon in wireless sensor networks. IEEE Trans. Parallel Distrib. Syst..

[18] Zhang Z., Yu F., Zhang Z. Collaborative localization algorithm for wireless sensor networks using mobile anchors. Proceedings of the IEEE Asia-Pacific Conference on Computational Intelligence and Industrial Applications (PACIIA).

[19] Song Z., Ma J., Dong M., Wang W., Gong X., Que X. Phone-Radar: Infrastructure-free Device-to-device Localization. Proceedings of the IEEE Vehicular Technology Conference (VTC).

[20] Jimenez A.R., Seco F., Prieto C., Guevara J. A comparison of pedestrian dead-reckoning algorithms using a low-cost MEMS IMU. Proceedings of the IEEE International Symposium on Intelligent Signal Processing (WISP).

[21] Chen W., Zhang X. A novel EMG-based stride length estimation method for pedestrian dead reckoning. Proceedings of the ION International Technical Meeting of the Satellite Division of the Institute of Navigation (ION GNSS).

[22] Bose A., Foh C.H. A practical path loss model for indoor WiFi positioning enhancement. Proceedings of the IEEE International Conference on Information, Communications and Signal Processing (ICICS).

[23] Isakov S.V., Zintchenko I.N., Rønnow T.F., Troyer M. (2015). Optimised simulated annealing for Ising spin glasses. Comput. Phys. Commun..

[24] Zheng Y., Xie X., Ma W.Y. (2010). GeoLife: A Collaborative Social Networking Service among User, Location and Trajectory. IEEE Data Eng. Bull..

[25] Constandache I., Gaonkar S., Sayler M., Choudhury R.R., Cox L. Enloc: Energy-efficient localization for mobile phones. Proceedings of the IEEE International Conference on Computer Communications (INFOCOM).

[26] Serrano P., Garcia-Saavedra A., Bianchi G., Banchs A., Azcorra A. (2015). Per-frame energy consumption in 802.11 devices and its implication on modeling and design. IEEE/ACM Trans. Netw..

[27] Yoon C., Kim D., Jung W., Kang C., Cha H. AppScope: Application Energy Metering Framework for Android Smartphone Using Kernel Activity Monitoring. Proceedings of the USENIX Annual Technical Conference (USENIX ATC).

[28] Song Z., Liu C.H., Wu J., Ma J., Wang W. (2014). Qoi-aware multitask-oriented dynamic participant selection with budget constraints. IEEE Trans. Veh. Technol..

[29] IBM ILOG CPLEX V12.6. http://www.ilog.com/products/cplex.

